# Efficacy of a Virtual Reality Rehabilitation Protocol Based on Art Therapy in Patients with Stroke: A Single-Blind Randomized Controlled Trial

**DOI:** 10.3390/brainsci14090863

**Published:** 2024-08-27

**Authors:** Gaetano Tieri, Marco Iosa, Antonio Fortini, Federica Aghilarre, Federico Gentili, Cristiano Rubeca, Tommaso Mastropietro, Gabriella Antonucci, Roberto De Giorgi

**Affiliations:** 1 Virtual Reality & Digital Neuroscience Lab, Department of Law and Digital Society, Unitelma Sapienza University, 00161 Rome, Italy; gaetano.tieri@unitelmasapienza.it; 2 IRCCS Santa Lucia Foundation, 00179 Rome, Italy; gabriella.antonucci@uniroma1.it; 3 Department of Psychology, University Sapienza of Rome, 00185 Rome, Italy; mastropietro.1815122@studenti.uniroma1.it; 4 Casa di Cura Nomentana Hospital, Fonte Nuova, 00013 Rome, Italy; antonio.fortini@nomentanahospital.it (A.F.); federica.aghilare@gmail.com (F.A.); federico.gentili@nomentanahospital.it (F.G.); rubecacristiano@gmail.com (C.R.); roberto.degiorgi@nomentanahospital.it (R.D.G.)

**Keywords:** neurorehabilitation, art therapy, neuroaesthetics, cerebrovascular accident, neurological rehabilitation

## Abstract

Background: Art therapy has a long history of applications in cognitive and motor rehabilitation. More recently, a growing body of scientific literature has highlighted the potential of virtual reality in neurorehabilitation, though it has focused more on the technology itself than on the principles adopted in digital scenarios. Methods: This study is a single-blind randomized controlled trial conducted on 40 patients with stroke, comparing conventional therapy (physical therapy for the upper and lower limbs, for posture and balance, cognitive therapy, occupational therapy, speech therapy, and specific therapy for swallowing, bowel, and bladder dysfunctions) to a protocol in which the upper limb physical therapy was substituted with art therapy administered by means of virtual reality exploiting the so-called Michelangelo effect. Results: After 12 sessions, patients in the virtual art therapy group showed a significantly greater improvement in independence in activities of daily living, as assessed by the Barthel Index (interaction of time and group: *p* = 0.001). Significant differences were also found in terms of upper limb muscle strength (Manual Muscle Test, *p* < 0.01) and reduction in spasticity (Ashworth scale, *p* = 0.007) in favor of the experimental group. In the virtual art therapy group, the effectiveness of the intervention was significantly correlated with patient participation (Pittsburgh Rehabilitation Participation Scale: R = 0.41), patient satisfaction (R = 0.60), and the perceived utility of the intervention by the therapist (R = 0.43). Conclusions: These findings support the efficacy of virtual art therapy leveraging the Michelangelo effect. Further studies should also focus on cognitive domains that could benefit from this type of approach.

## 1. Introduction

Virtual reality (VR) is one of the most promising technologies for neurorehabilitation. The main advantages of this technology are the ease of use, its safety, the low cost with respect to other technologies, its portability, and the embedded sensors that can provide informative data [1]. However, in many reviews, the efficacy of virtual reality has been evaluated by including non-immersive interactive video gaming systems into the meta-analysis [2,3], despite the fact that VR has a specific definition. In fact, VR has been defined as a high-end user–computer interface involving real-time stimulation and interactions of the subject embedded into a digital world through multiple sensorial channels in which the subject feels his/her presence, the ownership of the body of his/her avatar, and the possibility to interact with that world [4,5].

Another critical limitation of the existing literature is that the meta-analysis aggregated studies are based solely on VR hardware, disregarding software-specific content and rehabilitative principles. This approach conflates diverse VR applications, including gaming, physical and cognitive exercises, and real-life simulations.

A recent review highlighted the importance of task-specific training paradigms in VR for optimizing motor learning and facilitating skill transfer to obtain tangible enhancements in real-world functional abilities [6]. Another study emphasized the importance of defining the virtual task and the digital environment based on the rehabilitation objectives and patients’ needs, rather than focusing solely on the type of environment [7]. In fact, the type of exercises performed in the digital environment with multisensory feedback can lead to improvement in the patient’s motivation, engagement, and enjoyment [8]. A recent review summarized these findings, concluding that VR can improve patients’ compliance with treatment, ultimately increasing their level of functioning and quality of life [9]. In 2021, we tested the usability of VR for administering a protocol of art-therapy in which patients with stroke could paint a virtual canvas [10]. A novel aspect of this approach was the potential of VR to induce the illusion in patients that they were perfectly replicating iconic artworks, such as the Creation of Adam of Michelangelo or the Dance of Matisse. The proposed protocol combined the advantages of visual art fruition, mainly related to the wide arousal of brain networks activated by the vision of artistic masterpieces [11], and the active involvement typical of artistic creation [12].

This approach allowed us to discover the “Michelangelo effect”; i.e., healthy subjects and patients perceived less fatigue and were more accurate in their upper limb movements when they had the illusion of painting art masterpieces with respect to when they were simply asked to color a digital canvas, highlighting the importance of the artistic content within the required task [10].

In a previous pilot study, involving 20 patients with stroke in the subacute phase (10 treated with this virtual art-therapy protocol and 10 with conventional rehabilitation), we found a statistically significant higher improvement in patients treated with VR in terms of independence in activities of daily living (*p* = 0.021, assessed by Barthel Index). The improvements in upper limb force (Manual Muscle Test) and in reduction in spasticity (Ashworth Scale) only approached the statistically significant threshold (*p* = 0.063 and *p* = 0.055, respectively), suggesting the need to enroll more patients for obtaining more consistent results [13].

In the present study, we expanded the data collection by using the previous protocol [10], doubling the number of patients with stroke in the subacute phase.

The aim of this study was to assess the efficacy of the proposed virtual reality art-therapy protocol with respect to conventional therapy for patients with stroke. The secondary aim is to test whether the efficacy of VR is correlated with the active participation of patients or to a reduction in fatigue perception in the VR protocol.

## 2. Materials and Methods

### 2.1. Participants

The study was conducted in accordance with the ethical standards in the 2013 Declaration of Helsinki, and approved by the Local Ethical Committee. All patients signed written informed consent form. Inclusion criteria were: patients admitted to our hospitals for rehabilitation, clinical diagnosis of ischemic or hemorrhagic stroke confirmed by computerized tomography or magnetic resonance, age between 40 and 90, subacute phase of stroke (within 6 months from the acute event, when the rehabilitation could be more effective [14,15]), and capacity of performing voluntary movements, even if minimal, with the affected upper limb (Manual Muscle Test > 0). Exclusion criteria were: inability to comprehend and follow the therapist’s instructions (Mini Mental State Evaluation < 24); presence of risk of visual epilepsy, unilateral spatial neglect, severe comorbidities, and flaccid paralysis of the affected upper limb. Despite VR and 3D images so far appearing benign, light flashes, patterns, or color changes can provoke seizures [16], so we preferred to consider the risk of visual epilepsy as an exclusion criterion.

### 2.2. Study Protocol

This study consists in a single-blind randomized controlled trial (RCT) that completed the enrollment of patients of our previous pilot study [10] conducted in a single Italian hospital (Nomentana Hospital). Patients were randomly assigned to the experimental therapy group (EG) or conventional therapy group (CG) after their enrollment with an allocation ratio of 1:1. Randomization was performed using a computer-generated blocked random list, unveiling each allocation only after the enrolment of each patient.

Conventional therapy consisted of 3 rehabilitative sessions per day, each one of 1 h, 6 days per week, for one month (after which our protocol ended, but patients could continue their therapies). During these 3 daily hours, rehabilitation could include not only physical therapy for upper and lower limbs, posture, and balance, but also cognitive, occupational, and speech therapies, with specific therapy for swallowing, bowel, and bladder dysfunctions when clinically needed. The only difference for patients enrolled in experimental therapy group was that 3 weekly sessions of conventional upper limb physical therapy aiming at arm functional recovery were substituted by art therapy administered by virtual reality for a period of 4 weeks. The amount of time spent in rehabilitation, the frequency of therapy, its intensity, and the typologies of other daily therapies did not differ between the two groups.

**Figure 1:**
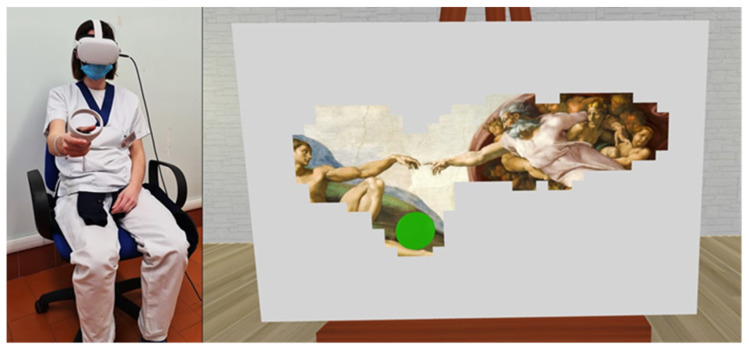
The figure illustrates a virtual art therapy example. The **left panel** depicts a person wearing a VR headset and holding the Oculus controller in her right hand. The **right panel** presents the first-person perspective within the virtual environment, where the virtual canvas is occluded by white pixels. The green spherical brush (placed in the same position of the joystick grasped by the participant) is used to remove the white pixels, revealing the underlying image.

During virtual art-therapy sessions, the patients comfortably sat on a chair wearing a Head-Mounted Display (Oculus Quest 2, Meta) and taking into his/her paretic hand the joystick which allowed him/her to control the virtual stimuli. The virtual environment, designed by using 3ds MAX 2018 and implemented in Unity 2018 game engine software with customized C# scripts, consisted of a room having, in the middle, a white canvas on an easel. The dimensions of the virtual area of the canvas were 40 cm × 60 cm. The subject saw in the virtual environment a spherical brush at the same position of the controller grasped by the patient with the affected hand. Patients were previously informed that the brush can color the canvas by touching it, forming a painting. Patients had the illusion to actually paint an artwork because the touch of the virtual brush delete white thin virtual pixels covering the whole surface of the canvas which occluded the visibility of the underlying painting. The target pixels were deleted when the subject moved the virtual brush keeping it in touch with the canvas, allowing to see a part of the underlying painting (a masterpiece of history of the art; see Figure 1 for an example). Patients performed different trials during the time dedicated to the rehabilitative sessions, unveiling in each trial a different famous painting associated to concept of beauty in previous studies (masterpieces of Michelangelo, Leonardo, Renoir, van Gogh, Caravaggio, etc. were used; conversely, abstract art was not included) [10,17]. Each trial was performed under the supervision of a trained physiotherapist who monitored that the patient executed the correct movements and maintained the correct posture according to his/her rehabilitation needs. As for conventional therapy, the assistance of the therapist was allowed when needed and the patient could have a rest if needed.

The CONSORT checklist of this study has been added as Supplementary Material.

### 2.3. Assessment

All patients were assessed at baseline (after enrolment) and 1 month later (at the end of this period of therapy) by a clinical assessor blind to the allocation group of the patient. The assessments were performed measuring the independence of the patient into the activities of daily living using the modified version of the Barthel Index (BI, primary outcome measure)) [18], the upper limb strength using the Manual Muscle Test (MMT) [19], and the spasticity of the upper limb using the Ashworth scale (AS) [20]. For the patients of EG, participation in each session of therapy was assessed using Pittsburgh Rehabilitation Participation Scale [21,22]. After each trial, the perceived fatigue was assessed by asking to the patient: “How tiring was this trial on a numeric rating scale ranging from 0 (no fatigue) to 10 (maximum possible fatigue)?” [13].

### 2.4. Statistical Analysis

Sample size was computed on the basis of our previous data [13]. Because the Barthel Index was already significant different between the two groups in our previous study, we evaluated the sample size by the effect size evaluated on the Manual Muscle Test scores (see the following paragraph 2.3 for assessment scales) with a result of 1.2. Setting the alpha level at 5%, the power of the test (1-beta) at 95%, and a possible drop-out rate of 25% on G-Power 3.1.9.7, the resulting sample size was 20 patients for each group. Data are reported in terms of mean and standard deviation for continuous measures, or percentage frequencies for nominal variables. The normality of data was tested using Kolgomorov–Smirnov analysis. For normally distributed variables, mixed analysis of variance was used to assess the effect of time (pre vs. post, within subject parameter), group (EG vs. CG), and their interaction. Effect size (ES) was computed using the partial eta-squared. For not-normally distributed variables, non-parametric Wilcoxon test for paired comparisons (pre vs. post) and Mann–Whitney U-test for unpaired comparisons between groups (EG vs. CG) were used. The efficacy of the interventions was computed in terms of effectiveness [23,24], computed as follows:

(1) $$Effectivness=\frac{Score\;Post-Score\;Pre}{Maximum\;of\;Scale-Score\;Pre}\times 100$$

The PRPS- and fatigue-scores were averaged (firstly, among trials for fatigue) among sessions for each patient, and their correlations with other clinical variables were computed using one-tailed Spearman coefficient (R). For all the analysis, the level of statistical significance for rejecting the null hypothesis was set at 5%.

## 3. Results

### 3.1. Comparison of the Experimental and Control Groups

Drop-out cases were recorded neither in the EG nor in the CG. Table 1 shows the demographical and clinical characteristics of the two groups at baseline. No significant differences were noted between groups, but, for spasticity, it was 0.9 points lower in the EG than in the CG.

**Figure 2:**
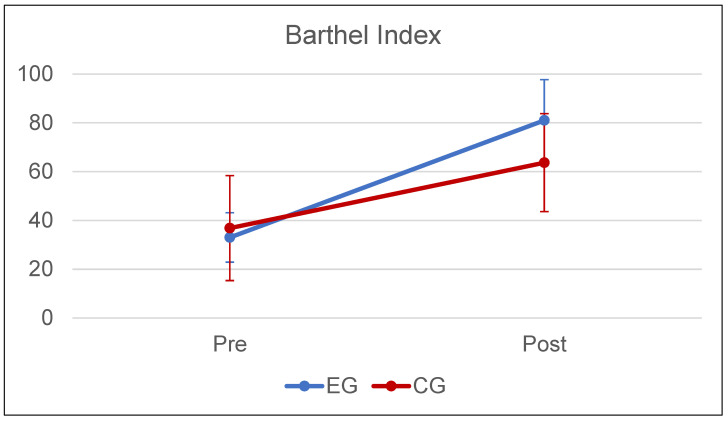
Mean ± standard deviation of Barthel Index pre- and post-treatment for Experimental Group (EG, in blue) and Control Group (CG, in red).

Figure 2 shows the primary outcome (Barthel Index) at baseline and after treatment. The mixed analysis of variance highlighted a significant effect of Time (pre vs. post: F(1,38) = 154, *p* < 0.001, ES = 0.802), a significant interaction Time*Group (F(1,38) = 12, *p* = 0.001, ES = 0.245), and a not-significant effect of Group (EG vs. CG: F(1,38) = 2, *p* = 0.158, ES = 0.052). A non-parametric test confirmed the significant differences of the BI-score post treatment (*p* = 0.003).

The effectiveness in terms of BI-score was 73 ± 22% in the EG and 39 ± 29% in the CG (*p* < 0.001).

**Figure 3:**
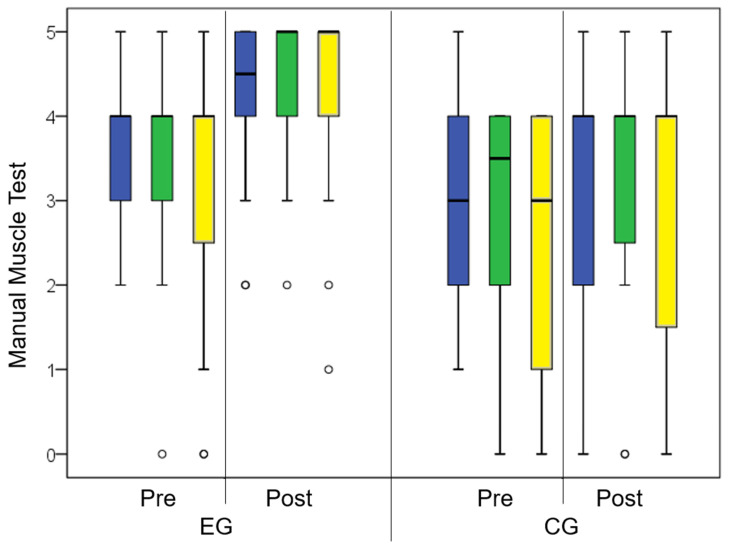
Box whiskers plot of Manual Muscle Score for shoulder abduction (blue bars), elbow flexion (green bars), and pinch (yellow bars) pre- and post-treatment for Experimental Group (EG, on the left) and Control Group (CG, on the right). The boxes represent the distance between 1st and 3rd quartiles and contain the medians (wide black lines), whereas the black whiskers represent 1.5 times the interquartile ranges, with values out of this range reported as circles.

After treatment, the scores of MMT (Figure 3) were also significant higher (u-test) in the EG than in the CG for shoulder abduction (*p* < 0.006), elbow flexion (*p* = 0.003), and pinch (*p* < 0.001). The Ashworth scale showed a lower spasticity in the EG than in the CG (*p* = 0.007).

### 3.2. Factors Influencing the Outcome in the Experimental Group

In the experimental group, the effectiveness was not significantly different between male and female patients (73 ± 23% vs. 72 ± 22%, *p* = 0.757), patients with left- or right-affected body side (66 ± 25% vs. 77 ± 20%, *p* = 0.181), and patients with ischemic or hemorrhagic stroke (73 ± 22% vs. 67 ± 25%, *p* = 0.736). In general, also for the other outcome scores, the experimental group did not show any differences related to these variables.

The mean value of participation was PRPS = 5.5 ± 0.5, that of fatigue was 2.2 ± 1.9, the mean value of patient’s satisfaction 9.2 ± 0.9, and that of the therapist’s perceived utility 9.7 ± 0.4 (PRPS had a maximum possible score of 6, whereas 10 was the maximum of the other variables).

Significant correlation was found between effectiveness in terms of BI and participation measured with PRPS averaged among sessions (R = 0.41, *p* = 0.036). Participation was found to be significantly correlated also with the utility of VR therapy perceived by the therapist (R = 0.43, *p* = 0.031) and with the level of satisfaction reported by each patient (R = 0.60, *p* = 0.002). Finally, the utility perceived by the therapist and the satisfaction reported by the patient were each other significantly correlated (R = 0.58, *p* = 0.004). Fatigue negatively but not significantly correlated with effectiveness, participation, the patient’s satisfaction, and the therapist’s utility perception.

## 4. Discussion

In the recent years, there has been a growing body of literature about the advancements of virtual reality and its use in different fields, from entertainment to scientific research, and from education to rehabilitation, including both physiotherapy and psychotherapy [25]. However, little attention has been given to the potential of virtual reality for art therapy.

Our previous pilot study [13] was conducted on twenty patients, ten per group, showing a significantly higher improvement in terms of the Barthel Index score and pinch strength in patients treated with the adjunction of virtual art therapy with respect to those who received additional conventional therapy. Neither the changes in the MMT total score nor in the Ashworth spasticity score achieved the significant threshold. According to the sample size calculation, we increased our samples by including 20 patients per group. As hypothesized, the improvements in MMT sub-scores and Ashworth scores were statistically significant. We should note the slight but significant difference in terms of spasticity in favor of the EG at baseline (0.9 point less on average) with respect to the CG. Before treatment, the EG showed a slightly higher upper limb functioning than the CG, but also a slight lower independence in the activities of daily living (4 points less on BI-score), but these differences were not statistically significant.

At the end of treatment, the art-therapy administered by virtual reality was more effective than conventional treatment, confirming the first hypothesis of this study.

A recent study investigated whether adding VR training into early rehabilitation may have substantial positive effects on patients with acute stroke, but it found only benefits on psychological health, specifically depression, but not muscle strength and functional recovery [26]. Conversely, we found significant effects in our study on the BI- and MMT-scores, and it could be related to the combination of VR and art therapy. Despite the fact that caution is needed for the reduced sample size of our study, this study suggests that a protocol of virtual art therapy exploiting the Michelangelo effect could be effective in the rehabilitation of patients with stroke.

The analysis conducted within the EG showed a significant correlation between the effectiveness of the treatment with the participation of the patient assessed by the therapist using the PRPS. In addition, the utility perceived by the therapist and the level of satisfaction perceived by the patient were significantly correlated with the assessed effectiveness on BI-score.

It is known that fatigue and exertion can be reduced during physiotherapy by listening to music [27], which could also improve muscle activity and motor movements [28]: our previous studies on the Michelangelo effect extend to virtual painting these positive effects ofart, which could have these benefits on rehabilitation [13,17]. In the present study, fatigue did not correlate with the efficacy of the intervention, but it should be noted that the therapists, during virtual art therapy (as well as during conventional therapy), adapted the level of difficulty of the exercises to the abilities of the patient. We assessed the patients’ fatigue, participation, and satisfaction in the EG with the aim of investigating how the Michelangelo effect may empower the efficacy of virtual art therapy. A limit of our study was that we did not assess these factors also in the CG, not allowing comparisons between groups. Then, we selected the independence in the activities of daily living assessed by the Barthel Index as the primary outcome, in line with many studies (as reported in a recent review [29]) and Italian guidelines for quantifying the efficacy of rehabilitation. A recent protocol proposes a multicenter study, with the Fugl–Meyer scale as primary outcome compared with multimodal brain imaging [30]. Our study was conducted on a single center, and neither the Fugl–Meyer scale nor functional imaging was included. Conversely, it could be very interesting to see the brain networks associated to the recovery obtained by means of virtual art therapy. We did not find significant differences between stroke on the right and left side, but this could be due to the reduced sample size of these subgroups in our study. In fact, we found an effectiveness of 77% in patients with the right side of the body affected by stroke, and 66% in those with left hemiparesis. The latter patients are those with a lesion in the right hemisphere of the brain, which is known to be more involved in aesthetic perception [31]. Furthermore, right hemispheric stroke may cause lower participation outcomes related to the little awareness of limitations [32]. Further studies should investigate the relationship between brain damages and the efficacy of virtual art therapy. According to these studies, another limit of our study is that important psychological aspects that may affect rehabilitation efficacy were not assessed. For example, it has been reported that participation in arts activities can reduce depression and improve quality of life, self-efficacy, and compliance with treatment in patients with stroke [33,34]. Virtual reality can be combined with art-therapy, reducing anxiety and social difficulties especially in adolescents, because they are “digital natives” [35], but the growing diffusion of digital technologies is also increasing the compliance to these approaches in elderly patients [36]. A recent review also reported that VR-based cognitive therapies may help patients with stroke in recovering their memory, executive functions, and overall cognitive functions [37]. Gao and colleagues also highlighted the importance of subgroup analyses in investigating how various moderating factors, such as the type of VR program, the duration of the intervention, and the stage of stroke recovery, may affect the relationship between VR-supported exercise therapy and related rehabilitative outcome [38].

According to the recent dual-task approach of rehabilitation, that combines motor and cognitive therapies, it could be important to also analyze the impact of art-therapy on cognitive domains. There are promising results about the exploitation of the Michelangelo effect on memory rehabilitation [39] and, more in general, on the use of digital technologies, such as virtual reality associated to complementary medicine [40], and also as visual art therapy associated to the recovery of cognitive functions [41]. This approach could also be combined with telerehabilitation [42] for the continuous care of patients with stroke also during chronic phase. In fact, another advantage of virtual reality is that this technique can easily be combined with other technologies for boosting their efficacy, such as transcranial magnetic stimulation [43] and robotic devices [44]. The fast diffusion of artificial intelligence (AI) may boost the use of digital technologies combined with art-therapies as a result of the easy-to-use generative tools of AI. It has been reported that AI chatbot interactions and AI-generated artworks may facilitate discussions about emotions, encourage self-expression through art creation, and provide cognitive–behavioral therapeutic advice in both verbal and visual ways [42]. Moreover, AI can be further implemented in our virtual scenario for adapting the level of difficulty for each patient and analyzing kinematics data in real time.

### Conclusions

The present study demonstrated that art-therapy combined with virtual reality, capitalizing on the Michelangelo effect in neurorehabilitation, may be an effective approach in enhancing independence in activities of daily living and upper limb muscle strength among stroke patients. The more the participation of patient in the therapy, the more the obtained benefit. Both the patient and therapist were satisfied with this type of intervention. We had previously assessed the usability of our protocol [17] and its feasibility in clinical settings [13]. The present findings showed that virtual art therapy could easily be integrated into daily clinical practice to the satisfaction of patients and therapists, leading to an increased participation of patients in their own therapy, leading to an improvement in its efficacy. Our study showed that 12 sessions of 1 h were sufficient to improve rehabilitation outcome as a result of the increased motivation obtained by virtual art therapy.

Further studies should investigate the long-term benefits for patients by including a follow-up assessment and also including the assessment and treatment of cognitive domains, for which art-therapy was shown to be effective [45,46], and it could be empowered by new technologies such as virtual reality and artificial intelligence.

## Figures and Tables

**Table 1:** Mean ± standard deviation or percentage frequency of demographical and clinical variables (H: hemorrhagic stroke; L: left body side; MMT: Manual Muscle Test) assessed at baseline for the two groups and the *p*-value of their comparisons (*t*-test for age, U-test for clinical scales, and chi-squared test for percentages).

Variable	Experimental Group	Control Group	*p*-Value
Age (years)	67 ± 10	69 ± 15	0.754
Gender (% females)	35%	45%	0.729
Type of stroke (%H)	20%	25%	0.841
Affected body side (%L)	40%	45%	0.404
Barthel Index	33 ± 10	37 ± 21	0.725
Ashworth scale	0.6 ± 0.7	1.5 ± 1.1	0.011
MMT shoulder	3.5 ± 1.0	2.9 ± 1.3	0.119
MMT elbow	3.6 ± 1.2	2.8 ± 1.5	0.120
MMT pinch	3.3 ± 1.6	2.4 ± 1.7	0.091

## Data Availability

The data that support the findings of this study are available from the corresponding author, M.I., upon reasonable request. The data are not publicly available due to privacy and ethical clinical restrictions.

## Supplementary Materials

The following are available online at https://www.mdpi.com/article/10.3390/brainsci14090863/s1, CONSORT checklist.
